# Characteristics and predictors of family accommodation in Turkish individuals with obsessive-compulsive disorder

**DOI:** 10.55730/1300-0144.5620

**Published:** 2023-02-11

**Authors:** Tacettin KURU, Selime ÇELİK ERDEN, Veysel DOĞAN, Kadir KARAKUŞ

**Affiliations:** Alanya Alaaddin Keykubat University Training and Research Hospital, Department of Psychiatry, Antalya, Turkey

**Keywords:** Obsessive-compulsive disorder, family accommodation, culture, anxiety, depression

## Abstract

**Background/aim:**

Family accommodation (FA) is associated with disease severity and response to treatment rates in patients with obsessive-compulsive disorder (OCD) and is therefore particularly important in this patient group. This study investigated the structure of FA and associated factors in a Turkish society sample.

**Materials and methods:**

The study was carried out with 92 patients diagnosed with OCD for at least 1 year, who applied to Alanya ALKU Training and Research Hospital psychiatry outpatient clinic between February 2021 and March 2022. Sociodemographic data form, Family Accommodation Scale-Patient Form (FAS-PF), Dimensional Obsessive-Compulsive Scale (DOCS), Beck Depression Inventory (BDI), Beck Anxiety Inventory (BAI) and Acceptance and Action Questionnaire (AAQ-II) were applied to all of the cases. Relatives filled out the Sociodemographic Data.

**Results:**

The mean age of the patients was 32.11 ± 11.56 years. Sixty-nine (75%) of the patients were women. Fifty (54.3%) patients were married. All participants reported FA behavior at least once in the previous week. FA exhibited no significant variation by sex (p = 0.679) or marital status (p = 0.256). Significant positive correlation was determined between DOCS-T (r = 0.370, p < 0.001), AAQ-II (r = 0.261, p = 0.013), BDI (r = 0.235, p = 0.024) and BAI (r = 0.342, p = 0.001) scores and the FAS-PF. In the regression analysis, only OCD disease severity predicted FA [(β = 0.295, p = 0.036, 95% confidence interval (95% CI) = 0.02 to 0.55)]. Higher FA scores were obtained in this study (23.93 ± 15.28) compared to previous research in Western societies (14.3 ± 15.2).

**Conclusion:**

Clinicians should consider FA in the examination of OCD patients. High FA values for both frequency and severity suggest that interventions directed toward FA may be associated with more positive outcomes in outpatient Turkish patients with OCD.

## 1. Introduction

Obsessive-compulsive disorder (OCD) is a disease characterized by obsessions and compulsions capable of causing marked disturbance in the life of the affected individual. Obsessions are unwanted thoughts, impulses and fantasies that frequently cause significant distress, while compulsions are repetitive behaviors or mental acts that the individual feels compelled to perform as a reaction to the obsession, or according to rules that must be strictly applied [1]. The lifetime prevalence in adults is approximately 2.3% [2]. The annual prevalence of OCD according to the Turkish Mental Health Profile Research is 0.5% [3]. Family accommodation (FA) is defined as the changes made in their daily routines by relatives of individuals with the disease for the purpose of reducing the distress experienced by the patient. Family members generally adapt by taking part in some aspects of the OCD patient’s rituals, through avoidance, or by altering their daily routines. For example, accommodation in OCD may assume the form of reassurance (providing repeated reassurance that the situation causing the anxiety will not arise) and participating in rituals (such as cleaning) or waiting for rituals to be completed. Although FA may be seen in association with many mental diseases, the first systemic studies were performed for patients with OCD and revealed that FA is a widespread phenomenon [4]. Evidence has shown that nearly 90% of family members adapt to the symptoms to some degree. Approximately 60% of families exhibit FA behavior on a daily basis [4,5]. Additionally, FA develops despite more than 80% of family members believing that the obsessions and compulsions are illogical and 66% not believing that FA can ameliorate the symptoms of OCD [6]. Similarly to compulsions, FA rapidly reduces anxiety in patients with OCD. This prevents the patient developing tolerance to anxiety and the habituation process. FA is thus included in the negative reinforcement cycle and contributes to poorer patient functioning, greater severity of OCD symptoms and increased family distress and maladjustment. In support of this theoretical perspective, extensive observational studies of OCD have shown that FA is associated with symptom severity [7,8]. FA is a negative predictor, not only of severity of disease, but also for treatment. High FA levels are associated with less response to treatment [9–11]. Findings from recent studies of FA have made increased FA one of the treatment objectives. Preliminary evidence supports the idea of the efficacy of brief familial interventions for reducing FA. Thompson et al. showed that intervention provided for family members in terms of FA was more beneficial in reducing the severity of symptoms in patients. Regression analyses showed that changes in FA explained an important part of subsequent variance in OCD symptoms [12]. To the best of our knowledge, only one study has examined FA in Turkish society [13]. That research examined patients with moderate-to-severe and refractory OCD who were either inpatients or outpatients for treatment. The present research is therefore the first study capable of representing the outpatient group in Turkish society. The primary purpose of this study was to investigate the nature of FA in patients with OCD in Turkish society. In light of the available literature data on FA and OCD, we expected patients with OCD to confirm significant rates of FA. Secondly, we examined the clinical correlates of FA with OCD symptom severity, depression, anxiety, and psychological inflexibility (PI). When the literature was examined, it was hypothesized that FA would demonstrate a significant positive association with the female sex. We investigated the extent to which obsessive-compulsive disorder severity, PI, anxiety and depression predict FA. The effect of OCD severity on FA is the most frequently and consistently repeated finding in the literature; therefore, we expected the result that OCD severity would predict FA in our study results. Furthermore, the literature presents data that anxiety predicts FA more than depression; thus, in consideration of this, we expected in our study that anxiety would still predict FA, when OCD severity was added to the analysis. To the best of our knowledge, no study has been conducted on the relationship between FA and PI. As a transdiagnostic factor, it was hypothesized that PI was significantly associated with FA and would predict FA.

## 2. Materials and methods

### 2.1. Participants and procedures

The study procedures were carried out in accordance with the Declaration of Helsinki. The study protocol was approved by the Alanya Alaaddin Keykubat University Clinical Research Ethical Committee (decision: 13.01.2021/01-02).

All participants who consecutively applied to our hospital’s outpatient clinic, accepted to participate in the study, did not meet the exclusion criteria, and entirely completed the forms were included in the study. Ninety-two patients aged over 18 who presented to the Alanya Alaaddin Keykubat University Training and Research Hospital outpatient clinic for treatment between February 2021 and March 2022 and diagnosed with OCD based on Diagnostic and Statistical Manual of Mental Disorders Fifth Edition (DSM-5) criteria, were included in the study. A diagnosis of OCD and Axis-I disorders comorbid with OCD and included in the exclusion criteria were confirmed using the Structured Clinical Interview for DSM-5 Disorders (SCID-I) [14]. Accordingly, three patients with schizophrenia and other psychotic disorders and two patients with bipolar disorder were not included in the study. Ninety-two relatives primarily responsible for the care of the patients and living with them (aged over 18, with the closest contact with the patient and most involved in treatment), such as a spouse, parent, other relative, or friend, were also included. Following the clinical evaluations, the patients and relatives completed the study scales. Patients and relatives with schizophrenia, bipolar mood disorder, mental disability, dementia, major cognitive deficit, severe and nonstable major medical disease, or current substance dependence (apart from nicotine), were excluded from the study.

### 2.2. Psychometric evaluations

#### 2.2.1. Dimensional Obsessive-Compulsive Scale (DOCS)

Developed by Abramowitz et al., this scale contains four distinct obsession-compulsion symptoms, each with its own general definitions and examples [15]. It measures the severity of each symptom dimension, including avoidance behavior. It consists of four dimensions—contamination, responsibility, unacceptable thoughts, and symmetry— each consisting of five items scored between 0 and 4. The reliability and validity of the Turkish language version were studied by Şafak et al. [16]. The Cronbach alpha value for the entire scale in the present study was 0.900.

#### 2.2.2. Family Accommodation Scale-Patient Form (FASPF)

The FAS-PF was developed by Wu et al. [17]. The reliability and validity of the Turkish language version were established by Çöldür EÖ [13]. It consists of nineteen items and four subdimensions—direct participation in and facilitation of OCD symptoms, avoidance of OCD triggers, adoption of the patient’s responsibilities, and alteration of personal responsibilities. The FAS-PF is a five-point Likert-type scale (1 = never, 5 = every day). Possible scores on the scale range from 19 to 95, higher scores indicating higher FA. The internal consistency coefficient in the present study was 0.860.

#### 2.2.3. Acceptance and Action Questionnaire (AAQ-II)

AAQ-II was developed by Bond et al. and the reliability and validity of the Turkish language version were established by Yavuz et al. [18,19]. The Turkish language version of AAQ-II exhibits good internal consistency, with a Cronbach alpha coefficient of 0.84. It is a seven-point Likert-type scale with questions being scored from 1 (never true) to 7 (always true), consisting of seven items. High scale scores are interpreted as indicating high psychological inflexibility (decreased psychological flexibility). The internal consistency coefficient in our study group was 0.897.

#### 2.2.4. Beck Depression Inventory (BDI)

The BDI is a 21-question multiple choice self-report scale measuring the severity of depression [20]. It measures the somatic, cognitive, impulsive, and emotional symptoms of depression. The mean internal consistency of the BDI is 0.86. Participants evaluate their symptom severity in the previous 2 weeks on a four-point Likert-type scale ranging from 0 to 3. High scores indicate an increased depressive mood. The validity and reliability of the BDI were studied by Hisli et al. [21]. The internal consistency coefficient in our study group was 0.906.

#### 2.2.5. Beck Anxiety Inventory (BAI)

The BAI is a 20-item self-report Likert-type inventory measuring the severity of anxiety [22]. The twenty-one items in the inventory are scored from 0 (not at all) to 3 (severely. Higher scores indicate a higher level of anxiety. The BAI has high internal consistency (α = 0.92). The reliability and validity of the Turkish language version were studied by Ulusoy et al. [23]. The internal consistency coefficient in our study group was 0.926.

### 2.3. Statistical analysis

The study data was analyzed using the SPSS statistical software (version 22.0; IBM Corp., Armonk, NY, USA). An a priori power analysis was conducted using G*Power version 3.1.9.7 to determine the minimum sample size required to test the study hypothesis. Considering previous studies and our own study parameters, regression analysis was planned with six possible predictors (age, disease duration, anxiety, depression, psychological inflexibility, and OCD severity). Results indicated the required sample size to achieve 95% power for detecting a medium effect (0.15), at a significance criterion of α = 0.05, N = 89 for our study analyses. Therefore, the resulting sample size of N = 92 was considered adequate to test the study hypotheses [24].

The normal distribution was checked by examining the histogram and Q-Q Plot graphics and skewness-kurtosis values. Skewness and kurtosis values for the scale results being within a range of ±1 was regarded as normal distribution [25]. Multicollinearity was examined using VIF values (<10) [26]. Relationships between variables were evaluated using Pearson’s correlation test. Independent samples t-test was performed to compare FAS-PV scores in sex and marital status. A multiple linear regression model was tested in which FA represented the dependent variable, whereas severity of OCD, anxiety, depression and psychological inflexibility the independent variables. A second regression model was also tested in the study, with FAS-PV as the dependent variable and DOCS subdimensions as the independent variables. p values < 0.05 were regarded as significant.

## 3. Results

Ninety-two patients and ninety-two relatives were included in the study. The mean age of the patient group was 32.11 ± 11.56 years. Women constituted 75% of the OCD patient group, and the majority of patients were married (54.3%). Spouses or partners represented 52.2% of the patient relatives (Table 1).

The most common obsessions in the patient group were contamination (76.1%), followed by symmetry (51.1%). The most common compulsions were checking (78.3%) and washing (77.2%).

Values for the patients’ age and psychometric measurement parameters are given in Table 2. All measurement results exhibited normal distribution, their skewness and kurtosis values remaining within a ±1 range. The variance inflation factor (VIF) values for independent variables ranged from 2.014 to 2.250. All the scales in the study group exhibited good internal consistency values (Table 2).

The mean FA scale value in the study group was 23.93 ± 15.28. The FA scores in this Turkish sample were significantly higher than in two studies performed using FAS-PV in North American samples (M = 14.34, SD = 12.87, n = 61), t = 4.042, p < 0.001, d = 0.67) and (M = 14.3, SD = 15.2, n = 108), t = 4.004, p <0.001, d = 0.57) [17,27].

All participants reported FA behavior at least once in the previous week (at least 1 point on the FA Scale). The most frequent FA behaviors in the study group were giving reassurance for obsessions (73%) and providing reassurance for compulsions (69%). The least frequent FA behaviors were changing work or school programs (21%) and making excuses or lying (29%) (Table 3).

Independent samples t-test was performed to compare FAS-PV scores in sex and marital status. There was no significant difference in FAS-PV scores for men (22.78 ± 17.70) and women (24.32 ± 14.50); t (90) = −0.42, p = 0.68. No significant difference was observed in terms of FAS-PV scores (one case of widowhood was included in the single group) between married (25.60 ± 14.76) and single (21.95 ± 15.81) individuals; t (90) = −1.14, p = 0.26. Age (p = 0.463) and duration of disease (p = 0.929) exhibited no significant association with FAS-PV scores. DOCS-T (p < 0.001) subscale scores were significantly positively correlated with FAS-PV scores. Similarly, total AAQ-II (p = 0.013), BDI (p = 0.024), and BAI (p = 0.001) scores exhibited significant positive correlation with FAS-PV scores (Table 4).

A multiple linear regression model was tested, in which the FA represented the dependent variable and AAQ-II, BDI, DOCS-T, and BAI scores the independent variables. Predictors accounted for significant variation in FA [R^2^ = 0.168, F(4.87) = 4.402, p = 0.003]. Only DOCS-T results exhibited a significant predictive effect on FA (β = 0.295, p = 0.036, 95% confidence interval (95% CI) = 0.02 to 0.55 ) (Table 5).

A regression model was tested, with FAS-PV as the dependent variable and DOCS subdimensions as independent variables. In the model, only DOCS-Contamination results exhibited a significant predictive effect on FA (β = 0.251, p = 0.027, 95% CI = 0.08 to 1.35) (Table 6).

## 4. Discussion

The primary aim of this study was to investigate the clinical characteristics of FA and associated clinical and sociodemographic variables in an adult sample of patients with OCD in Turkish society. In concurrence with previous studies, we first observed that FA is a widespread phenomenon in the relatives of patients with OCD. FA behavior rates greater than 90% have been reported in previous research [28]. In the present study, all participants exhibited FA behavior at least once a week (scoring at least one point on any item on the scale).

The mean FAS-PV score of our study group is significantly higher than previous studies performed using the FAS-PV. Low FAS-PV values were reported in the original scale study conducted with a North American sample, as well as in another, more extensive study performed with the same method (FAS-PV) [17,27]. There has only been one study in the Turkish population, which was performed with treatment-resistant OCD patients and reported higher FA values than in the present study [13]. The high FA value in that study was interpreted as being potentially associated with a treatment-resistant group. The high FA results obtained in an outpatient group in the present study suggest that this result is not associated solely with the presence of a treatment-resistant group and may be the result of various culture-specific factors.

Cultural values and norms affect caregiving experiences, the cultural determinants of caregiving responsibilities or the caregiving hierarchy, and the values and norms underlying the decision to provide care [29]. High FA was reported in studies using FAS-PV in Chinese society, and this result was interpreted to be related to family-centered and collectivist cultural structure [30].The most frequently observed FA behaviors in the present research were the provision of reassurance for obsessions and compulsions. Similar to the results of our study, in previous studies using the same measurement method, waiting for the affected person to complete compulsive behaviors and providing reassurance regarding OCD-related concerns were the most frequently reported specific behaviors [17,27]. These behaviors may have been more common than other accommodation behaviors because they were passive and did not require action.

FA was not associated with sex or age in this study. Inconsistent results have emerged from previous studies. Some have reported an association between increased FA and female sex and increasing age, while others have determined no correlation with these parameters [27,31]. Close emotional ties and interdependence are common in Turkey. There is evidence that even older parents with independent incomes prefer to live with or close to their adult children, rather than living on their own, and close emotional bonds continue between generations. Thus, the shift in family interaction is toward sustained emotional interdependence [32]. This dominant structure in the Turkish family may mask the effect of variables such as sex and age. The results of our study did not reveal a significant relationship between disease duration and FAS-PV scores; similar results have been reported in previous studies [33,34]. These findings may be a reflection of the chronic and fluctuating course of OCD and that its severity does not inevitably have to increase continuously in this process. It should be noted that starting treatment at any stage of the disease process may affect both the severity of symptoms and the FA behavior of their relatives. However, the lack of untreated disease duration information in our study makes it difficult to interpret this result. As expected, significant positive correlation was observed in this study between FA and severity of OCD (total DOCS score), depression, anxiety, and psychological inflexibility. Inconsistent results concerning the relationship between depression and anxiety and FA have been reported in the literature [17,27,30,34]. In addition, severity of disease emerged as a significant predictor of FA at regression analysis. A recent metaanalysis also reported that a powerful prediction of FA by the severity of disease was the most consistent finding in the literature [35]. However, the direction of this relationship remains unclear due to the cross-sectional nature of this and previous studies. High disease severity precipitates helping feelings in caregivers and may result in greater exhibition of these behaviors. However, FA behavior may lead to the persistence and exacerbation of the disease, by preventing the patient from being exposed to and witnessing the results.

When the relationship between OCD symptom subdimensions and FA was examined, we found that only the contamination subdimension predicted FA, in line with previous studies [33]. The reasons for this result may be that cleaning compulsions are usually time-consuming or that they affect more noticeably the common living spaces with other individuals.

None of the other parameters, such as anxiety, depression, or psychological inflexibility, predicted FA and these results are consistent with the previous literature. A recent review study reported that anxiety and depression levels have no predictive role for FA in studies of adult OCD patients [35]. We encountered no previous studies of the effect on FA of psychological inflexibility, a transdiagnostic factor. Contrary to expectation, psychological inflexibility did not predict FA in this study. Psychological inflexibility is regarded as one of the basic pathologies underlying psychopathology and its mediating role in numerous psychological problems has been well demonstrated [36,37]. More extensive studies are therefore now warranted to investigate the mediating role of psychological inflexibility in FA.

There are a number of limitations to this study. The first is that it was performed in a single center. Our study results are particularly important for future studies comparing FA in patients with OCD between Turkish and other cultures. However, culture is a complex phenomenon. Different subcultures and economic conditions may produce different results in different regions in the same geographical area. Our results therefore require careful interpretation. In addition, psychological parameters concerning patient relatives were not evaluated in this study. The cross-sectional nature of the study should also be taken into account when interpreting the results. In our study, insight was not examined in OCD patients; therefore, the effect of insight on FA and other measurement parameters could not be reported. The possible reasons for refusal to participate among the patients who declined to do so (severity of OCD or specific obsessions) could also not be tested, and the possibility of selection bias should therefore also be taken into consideration.

In conclusion, FA behavior is a widespread phenomenon in Turkish families. Greater FA was observed among Turkish patient relatives than in studies involving Western populations. There is a significant positive correlation between FA and OCD severity (total DOCS score), depression, anxiety, and PI. We also found that OCD disease severity and contamination subdimension of OCD were significant predictors of FA. Notably, FA was not correlated with sex or age in our study.

### 4.1. Clinical implications

Clinicians should consider FA in the examination of OCD patients and they should be aware that the higher probability of FA is in patients with contamination subdimension symptoms. Studies on the effect of adding intervention on FA to treatment on treatment outcomes have been increasing recently. Evidence suggests that therapeutic interventions for FA in OCD patients positively affect treatment outcomes [12]. Therefore, approaches aiming to reduce FA by developing behavioral models that include FA seem valuable to achieve therapeutic goals. There is no study yet in this area in Turkish OCD patients. Considering the high FA results, we can assume that clinical intervention to FA in Turkish OCD patients may be associated with positive outcomes; however, intervention studies are needed to support this assumption.

## Figures and Tables

**Table 1:** Descriptive statistics of sociodemographic variables of patients/caregivers.

Patients		N-Mean	%-SD
Age (Year)		32.11	11.56
Sex	Male	23	25.0%
	Female	69	75 %
Marital status	Single	41	44.6%
	Married	50	54.3%
	Widowed	1	1.1%
Education	Elementary	26	28.3%
	High school	34	37.0%
	College/university	32	34.8%
Relatives			
Age (Year)		43.28	11.26
Sex	Male	50	54.3%
	Female	42	45.7%
Proximity	Parent	31	33.7%
	Spouse-partner	48	52.2%
	Other	13	14.1%
Marital status	Single	11	12.0%
	Married	79	85.9%
	Widowed-separated	2	2.2%
Education	Elementary	38	41.3%
	High school	22	23.9%
	College/university	32	34.8%
	Total	92	100

**Table 2:** Patients’ ages and study parameter results.

	Mean ± SD (Min–max)	Skew	Kurt	*α*
Age (Year)	32.11 ± 11.56 (18–68)	–	–	–
Years since onset of disease	6.48 ± 5.85 (1–25)	–	–	–
FAS-PV	23.93 ± 15.28 (1–76)	0.536	−0.558	0.860
DOCS-Contamination	10.47 ± 5.34 (0–20)	−0.262	−0.786	0.836
DOCS-Responsibility	9.04 ± 5.67 (0–20)	0.017	−1.117	0.893
DOCS-Unacceptable thoughts	10.18 ± 5.99 (0–20)	−0.081	−1.162	0.920
DOCS-Symmetry	7.74 ± 5.22 (0–20)	0.177	−0.564	0.880
DOCS Total score	37.43 ± 15.78 (9–71)	0.184	−0.769	0.900
AAQ-II	32.03 ± 11.64 (7–49)	−0.268	−0.863	0.897
BDI	24.40 ± 12.18 (0–47)	0.033	−0.771	0.906
BAI	21.57 ± 14.03 (0–53)	0.478	−0.912	0.926

AAQ-II: Acceptance and Action Questionnaire-II, DOCS: Dimensional Obsession-Compulsion Scale, BDI: Beck Depression Inventory, BAI: Beck Anxiety Inventory, FAS-PV: Family Accommodation Scale-Patient Version. *α*: Cronbach’s alpha. Skew: Skewness, Kurt: Kurtosis.

**Table 3:** Frequencies of FA items in the OCD sample.

Items	None/never *n* (%)	One day a week *n* (%)	2–3 days a week *n* (%)	4–6 days a week *n* (%)	Every day *n* (%)	Mean (SD)
1.	25 (27.2)	13 (14.1)	19 (20.7)	12 (13.0)	23 (25.0)	1.95 (1.54)
2.	29 (31.5)	13 (14.1)	18 (19.6)	8 (8.7)	24 (26.1)	1.84 (1.59)
3.	41 (44.6)	13 (14.1)	13 (14.1)	5 (5.4)	20 (21.7)	1.46 (1.61)
4.	50 (54.3)	9 (9.8)	12 (13)	4 (4.3)	17 (18.5)	1.23 (1.58)
5.	60 (65.2)	8 (8.7)	12 (13)	3 (3.3)	9 (9.8)	0.84 (1.34)
6.	56 (60.9)	6 (6.5)	9 (9.8)	3 (3.3)	18 (19.6)	1.14 (1.61)
7.	50 (54.3)	6 (6.5)	15 (16.3)	9 (9.8)	12 (13)	1.21 (1.50)
8.	49 (53.3)	11 (12)	10 (10.9)	7 (7.6)	15 (16.3)	1.22 (1.55)
9.	69 (75)	8 (8.7)	7 (7.6)	2 (2.2)	6 (6.5)	0.57 (1.15)
10.	41 (44.6)	12 (13)	9 (9.8)	6 (6.5)	24 (26.1)	1.57 (1.69)
11.	42 (45.7)	6 (6.5)	13 (14.1)	3 (3.3)	28 (30.4)	1.66 (1.75)
12.	47 (51.1)	7 (7.6)	14 (15.2)	12 (13)	12 (13)	1.29 (1.52)
13.	33 (35.9)	9 (9.8)	14 (15.2)	9 (9.8)	27 (29.3)	1.87 (1.68)
14.	71 (77.2)	6 (6.5)	9 (9.8)	2 (2.2)	4 (4.3)	0.50 (1.05)
15.	41 (44.6)	5 (5.4)	13 (14.1)	9 (9.8)	24 (26.1)	1.67 (1.70)
16.	37 (40.2)	10 (10.9)	7 (7.6)	11 (12)	27 (29.3)	1.79 (1.73)
17.	54 (58.7)	9 (9.8)	8 (8.7)	9 (9.8)	12 (13)	1.09 (1.50)
18.	79 (85.9)	3 (3.3)	2 (2.2)	5 (5.4)	3 (3.3)	0.37 (1.00)
19.	66 (71.7)	7 (7.6)	8 (8.7)	4 (4.3)	7 (7.6)	0.68 (1.26)

**Items;** 1: Reassurance (obsession), 2: Reassurance (compulsion), 3: Waited due to compulsions, 4: Participated in compulsions, 5: Facilitated compulsions, 6: Provided items, 7: Facilitated avoidance, 8: Helped with decisions, 9: Helped with personal tasks, 10: Prepared food, 11: Family/household responsibilities, 12: Avoided talking, 13: Stopped from doing things, 14: Made excuses/lied, 15: Tolerated, 16: Unusual conditions, 17: Cut back on leisure activities, 18: Changed work/school schedule, 19: Family responsibilities.

**Table 4:** Relationships between family accommodation and the participants’ sociodemographic and clinical characteristics.

	Pearson’s r (p-value)
Age	0.077 (0.463)
Duration of disease	0.009 (0.929)
DOCS-Contamination	0.313** (0.002)
DOCS-Responsibility	0.283** (0.006)
DOCS-Unacceptable thought	0.228* (0.029)
DOCS-Symmetry	0.228* (0.029)
DOCS-Total score	0.370** (< 0.001)
AAQ-II	0.261* (0.013)
BDI	0.235* (0.024)
BAI	0.342** (0.001)

Pearson correlation coefficients, AAQ-II: Acceptance and Action Questionnaire-II, DOCS: Dimensional Obsessive-Compulsive Scale, BDI: Beck Depression Inventory, BAI: Beck Anxiety Inventory, FAS-PV: Family Accommodation Scale-Patient Version.

** Correlation significant at the 0.01 level,

* Correlation significant at the 0.05 level.

**Table 5:** Multiple linear regression analysis for variables predicting family accommodation.

Predictor	B	SE (B)	β	t	p-value
Constant	10.083	4.625		2.180	0.032
BDI	−0.197	0.203	−0.157	−0.969	0.335
BAI	0.259	0.151	0.238	1.713	0.090
AAQ-II	0.074	0.193	0.056	0.381	0.704
DOCS-T	0.286	0.134	0.295	2.128	0.036

AAQ-II: Acceptance and Action Questionnaire-II. DOCS-T: Dimensional Obsessive-Compulsive Scale-Total. BDI: Beck Depression Inventory. BAI: Beck Anxiety Inventory. FAS-PV: Family Accommodation Scale-Patient Version. Dependent variable: FAS-PV.

**Table 6:** Multiple regression analysis of DOCS subdimensions for family accommodation.

Predictor	B	SE (B)	β	t	p-value
Constant	9.272	4.008		2.313	0.023
DOCS-Contamination	0.717	0.320	0.251	2.243	**0.027**
DOCS-Responsibility	0.540	0.303	0.200	1.782	0.078
DOCS-Unacceptable thoughts	0.180	0.292	0.070	0.615	0.540
DOCS-Symmetry	0.057	0.347	0.019	0.164	0.870

DOCS: Dimensional Obsessive-Compulsive Scale. Dependent Variable: FAS-PV: Family Accommodation Scale- Patient Version.
